# Revealing the Anti-Tumor Effect of Artificial miRNA p-27-5p on Human Breast Carcinoma Cell Line T-47D

**DOI:** 10.3390/ijms13056352

**Published:** 2012-05-23

**Authors:** Chien-Wei Tseng, Hsuan-Cheng Huang, Arthur Chun-Chieh Shih, Ya-Ya Chang, Chung-Cheng Hsu, Jen-Yun Chang, Wen-Hsiung Li, Hsueh-Fen Juan

**Affiliations:** 1 Department of Life Science, Institute of Molecular and Cellular Biology, National Taiwan University, Taipei 106, Taiwan; E-Mails: angus8921@gmail.com (C.-W.T.); b90206002@ntu.edu.tw (Y.-Y.C.); chung8421@yahoo.com.tw (C.-C.H.); jerris2004@gmail.com (J.-Y.C.); 2 Institute of Biomedical Informatics, Center for Systems and Synthetic Biology, National Yang-Ming University, Taipei 112, Taiwan; E-Mail: hsuancheng@ym.edu.tw; 3 Institute of Information Science, Research Center for Information Technology Innovation, Academia Sinica, Taipei 115, Taiwan; E-Mail: arthur@iis.sinica.edu.tw; 4 Biodiversity Research Center and Genomics Research Center, Academia Sinica, Taipei 115, Taiwan; 5 Department of Ecology and Evolution, University of Chicago, Chicago, IL 60637, USA

**Keywords:** miR P-27-5p, exon array, cyclin-dependent kinase 4, cell cycle, breast cancer, retinoblastoma protein

## Abstract

microRNAs (miRNAs) cause mRNA degradation or translation suppression of their target genes. Previous studies have found direct involvement of miRNAs in cancer initiation and progression. Artificial miRNAs, designed to target single or multiple genes of interest, provide a new therapeutic strategy for cancer. This study investigates the anti-tumor effect of a novel artificial miRNA, miR P-27-5p, on breast cancer. In this study, we reveal that miR P-27-5p downregulates the differential gene expressions associated with the protein modification process and regulation of cell cycle in T-47D cells. Introduction of this novel artificial miRNA, miR P-27-5p, into breast cell lines inhibits cell proliferation and induces the first “gap” phase (G1) cell cycle arrest in cancer cell lines but does not affect normal breast cells. We further show that miR P-27-5p targets the 3′-untranslated mRNA region (3′-UTR) of cyclin-dependent kinase 4 (CDK4) and reduces both the mRNA and protein level of CDK4, which in turn, interferes with phosphorylation of the retinoblastoma protein (RB1). Overall, our data suggest that the effects of miR p-27-5p on cell proliferation and G1 cell cycle arrest are through the downregulation of CDK4 and the suppression of RB1 phosphorylation. This study opens avenues for future therapies targeting breast cancer.

## 1. Introduction

microRNAs (miRNAs) cause mRNA degradation or translation suppression of their target genes. Previous studies have shown that miRNAs play crucial roles in tumorigenesis by targeting the mRNAs of oncogenes or tumor suppressors [1,2]. Many reports indicate that miRNA expression is altered in tumor tissues, suggesting that these miRNAs could be potential markers for detection and prognosis in human cancers [3,4]. Because of the critical role of miRNAs as regulators of cell fate, analyzing and manipulating miRNAs within cancer cells may provide powerful avenues for diagnosis, prognosis, drug discovery, and therapeutics [5]. miRNA P-27-5p (miR P-27-5p) is a novel miRNA we recently discovered in breast cancer cells [6]. In this study, we investigate why this artificial miRNA can suppress breast cancer cell growth.

Microarray is a powerful high-throughput technique for determining changes in global gene expressions in functional genomics [7,8]. It can measure expression of tens of thousands of discrete sequences in a single array [9]. The exon array is useful for exon-level expression profiling at the whole-genome scale on a single array [10]. The oligonucleotide probes of exon arrays greatly differ from those of conventional 3′ expression arrays in their design, density, and coverage. This technique has been used for novel genes discoveries [11], gene function determination, drug evaluation, pathway dissection, and clinical sample classification [9,12].

The G1/S cell cycle checkpoint controls the passage of eukaryotic cells from G1 into the DNA synthesis phase (S). Two cell cycle kinases, cyclin-dependent kinase 4/6 (CDK4/6)-cyclin D [13–15] and CDK2-cyclin E [16], and the transcription complex that includes retinoblastoma protein (RB1) and E2F are essential in controlling this checkpoint [17]. CDK4, a member of the Ser/Thr protein kinase family, binds cyclin D and subsequently phosphorylates RB1, leading to cell cycle regulation, which is thought to occur through hyperphosphorylation-induced release of the E2F transcription factors from the large pocket [17]. A breakdown in the regulation of this cycle can lead to out-of-control growth and contribute to tumor formation. Recently, many inhibitors specifically targeted to CDK4 have been developed for the treatment of breast cancer [14,15].

In this study, we show that miR P-27-5p inhibits the growth of breast cancer cells and induces cell cycle arrest at the G1 phase. Exon array, Western blot, real-time polymerase chain reaction (PCR), and luciferase report analyses have revealed that miR P-27-5p targets *CDK4*. Our observations indicate that miR P-27-5p inhibits cancer cell proliferation and triggers G1 cell cycle arrest by targeting *CDK4* and suppressing phosphorylation of RB1.

## 2. Results and Discussion

### 2.1. miR P-27-5p Downregulates Gene Expressions Associated with Cell Growth, Cell Cycle, and Phosphorylation in T-47D Cells

In addition to translation suppression, miRNAs cause mRNA degradation of their target genes; the changes at the mRNA level can be detected by microarray experiments [18,19]. We used a high-throughput exon array to identify the miR P-27-5p–regulated genes and potential targets. The results of exon array data have been submitted to the GEO database, and the series record is GSE28657.

**Figure 1:**
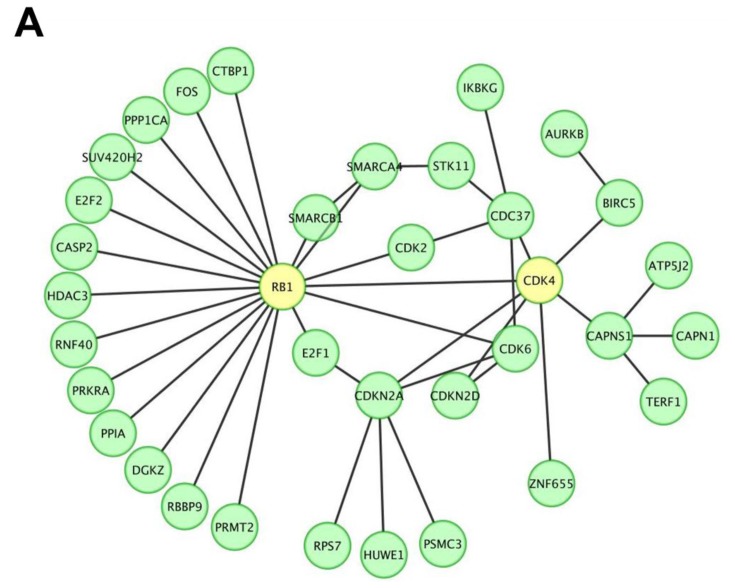

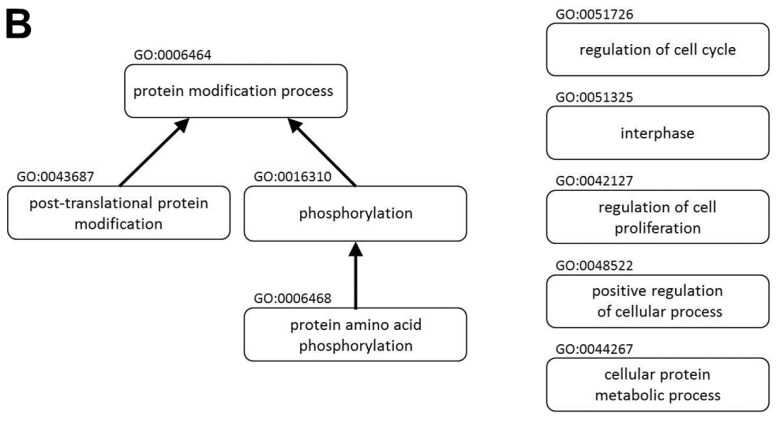
A protein-protein interaction network and the biological functions regulated by miR P-27-5p in T-47D cells. Gene expression profiles were determined using exon arrays. (**A**) The significantly differentially expressed proteins in miR P-27-5p–overexpressing tumor cells were used to construct the protein-protein interaction (PPI) network; (**B**) All proteins in the network were further analyzed for clustering of functional profiles by using BiNGO. It uncovered key functional relationships, particularly cell proliferation and cycle and phosphorylation.

To screen and investigate the gene expression profile and possible biological functions of miR P-27-5p regulation in breast cancer T-47D cells, we used our previously developed approach [20,21]. There were 2590 genes with a significant downregulated change in expression level between control and miR P-27-5p–transfected T-47D cells (Supporting Information Table S.1). These downregulated expressed genes were used to construct the protein-protein interaction (PPI) network. The human protein interaction network (PIN) was downloaded from the Human Protein Reference Database [22], and only the largest connected component was studied. The differentially downregulated expressed proteins in the miR P-27-5p–related network was further predicted by functional enrichment analysis. Among the PPI networks, we found that CDK4 and RB1 were involved in the network and the RB1 interacted with 19 proteins including CDK4 (Figure 1A).

All proteins in the network were further analyzed for clustering of functional profiles using BiNGO (*p* < 0.001). BiNGO [23,24], a Cytoscape [24] plug-in, was used to determine which Gene Ontology (GO) terms were significantly overrepresented (the hypergeometric test, Benjamini and Hochberg False Discovery Rate correction, *p* ≤ 0.001) in miR P-27-5p–related networks. Key functional relationships were revealed, including the protein modification process, protein amino acid phosphorylation, phosphorylation, regulation of cell proliferation, posttranslational protein modification, the cellular protein metabolic process, positive regulation of cellular process, interphase, and regulation of cell cycle (Figure 1B and Table 1).

### 2.2. miR P-27-5p Overexpression Inhibits the Growth of Breast Cancer Cells

**Figure 2:**
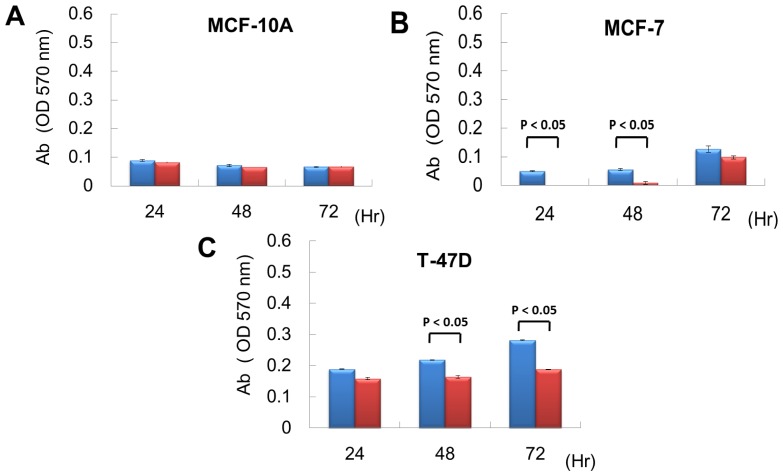
Inhibitory effect of miR P-27-5p on cell proliferation. The breast normal cells MCF-10A (**A**) and cancer cells MCF-7 (**B**) and T-47D (**C**) were transfected with 60 nM miR P-27-5p (red bar) and NC mimetics (blue bar). Cell viability was determined using an MTT assay. The relative cell proliferation was examined at the indicated time points by MTT. The absorbance of MTT by each sample was recorded at 570 nm after staining. The error bar shows SE for three independent experiments.

**Figure 3:**
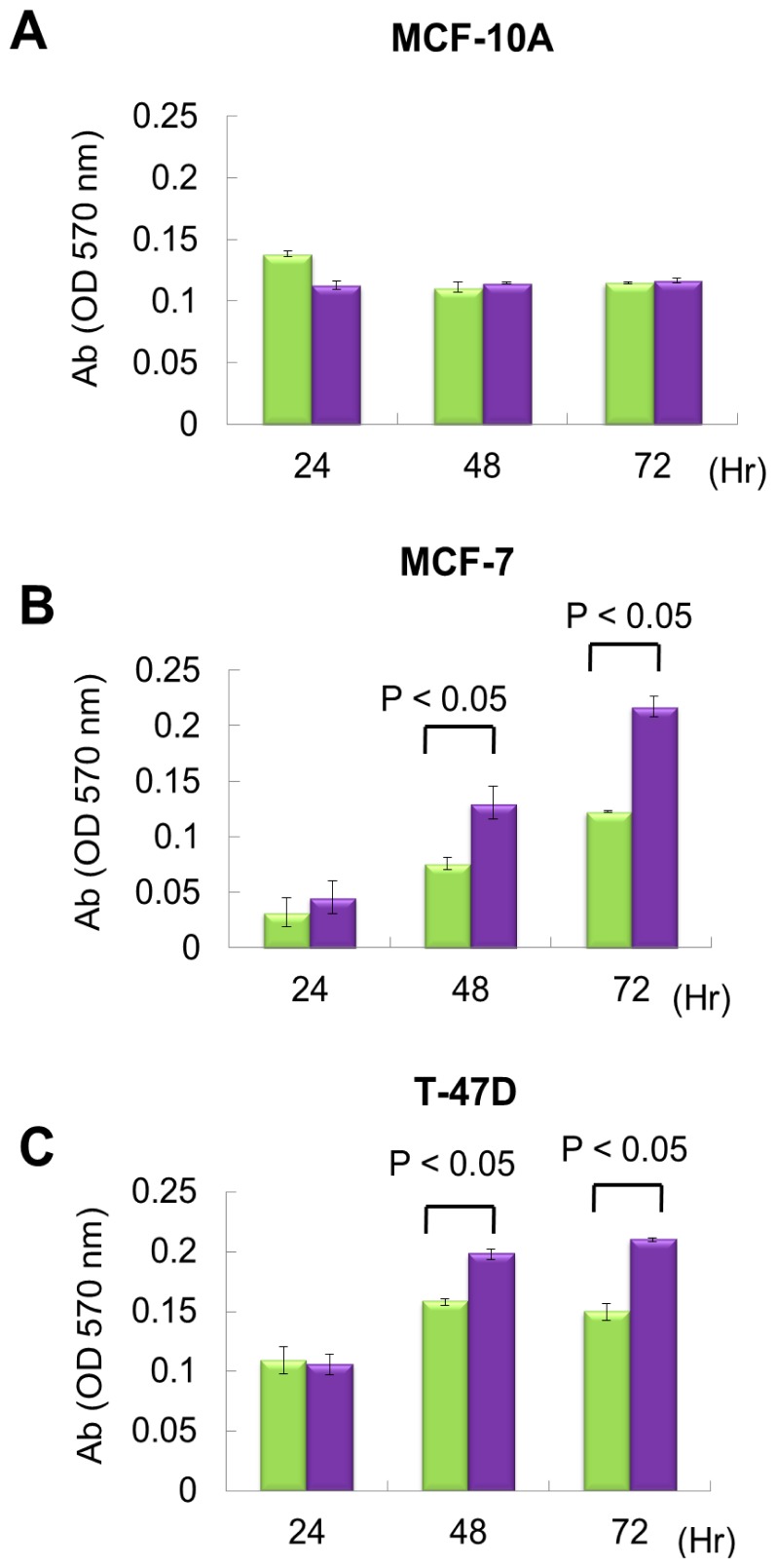
The effect of downregulation of miR P-27-5p on cell proliferation. The breast normal cells MCF-10A (**A**) and cancer cells MCF-7 (**B**) and T-47D (**C**) were transfected with 60 nM antisense miR P-27-5p (purple bar) and NC (green bar), respectively. Cell viability was determined using an MTT assay. The relative cell proliferation was examined at the indicated time points by methylthiazoletetrazolium (MTT). The absorbance of MTT by each sample was recorded at 570 nm after staining. The error bar shows SE for three independent experiments.

From exon array data, network and functional enrichment analysis, we found that miR P-27-5p overexpression could downregulate the gene expression levels involved in cell proliferation. To evaluate the effect of miR P-27-5p on cell growth, a methylthiazoletetrazolium (MTT) cell proliferation assay was used as described above. MCF-10A, MCF-7 and T-47D were transfected with miR P-27-5p mimics or negative control (NC). MTT assays were performed at 24 h, 48 h, and 72 h. As shown in Figure 2, transfection of miR P-27-5p into cell lines significantly inhibited cell proliferation of breast cancer cells MCF-7 and T-47D, but not the normal cells MCF-10A. Additionally, we found that transfection of antisense miR P-27-5p into cancer cells promoted cell proliferation (Figure 3). These results indicate that miR P-27-5p has an adverse effect on breast cancer cell proliferation.

### 2.3. miR P-27-5p Induces G1 Arrest in Breast Cancer Cells

**Figure 4:**
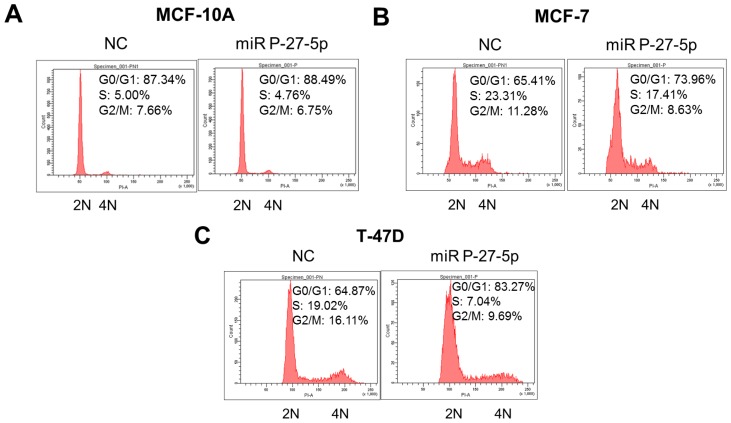
G0/G1 arrest by miR P-27-5p. The breast normal cells (MCF-10A) (**A**), and cancer cells MCF-7 (**B**) and T-47D (**C**) were transfected with miR P-27-5p and NC mimetics (100 nM) for 48 h. Then, cell cycle distributions of these cells were analyzed by flow cytometry. 2N: cells with diploid DNA content; and 4N: cells with tetraploid DNA content. The experiments were performed in triplicates.

In addition to cell proliferation, miR P-27-5p downregulates the genes involved in the cell cycle. To investigate the effect of miR P-27-5p on cell cycle progression of breast normal/cancer cells, their DNA contents were analyzed by flow cytometry, and the derived data were used to investigate the phase distribution of the cell cycle. Cells with 2 n and 4 n DNA content correspond to the G0/G1 and G2/M phases, respectively. Cells with a DNA content between 2 n and 4 n correspond to the S phase. As shown in Figure 4, after transfection with miR P-27-5p, the DNA percentage of the G0/G1 phase was increased from 65.41% to 73.96% in MCF-7 cells and from 64.87% to 83.27% in T-47D cells, but no significant change in MCF-10A cells was noted. These results show that miR P-27-5p induces G1 cell cycle arrest in breast cancer cells but not in normal cells.

### 2.4. Down-Regulation of CDK4 by miR P-27-5p at mRNA and Protein Levels via Direct Targeting 3′-UTR

**Figure 5:**
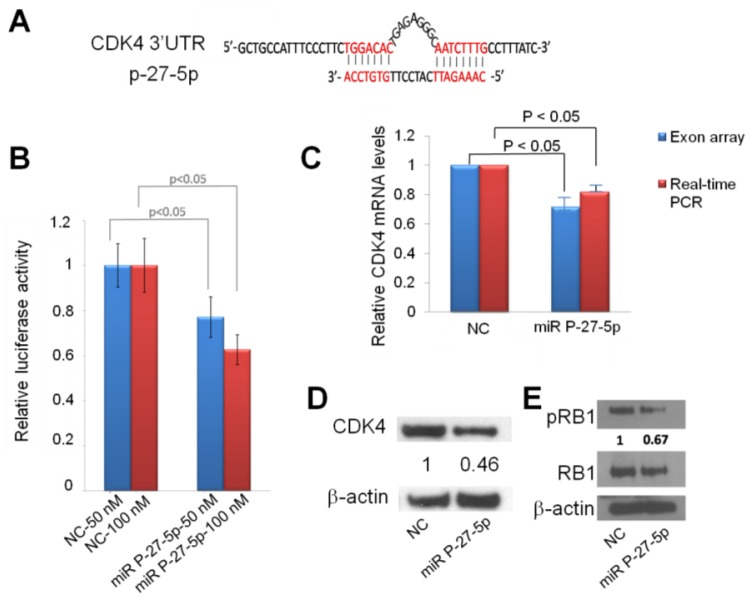
Down-regulation of *CDK4* by miR P-27-5p at the mRNA and protein levels via direct targeting of 3′-UTR. (**A**) The sequences of *CDK4* 3′-UTR and miR P-27-5p. (**B**) The effect of miR 27-5p on the expression of *CDK4* by using luciferase assays. Cells were cotransfected with miR P-27-5p duplex or NC with *CDK4* 3′-UTR. The reporter assays shown in this study were based on data averaged from at least three independent transfections. (**C**) *CDK4* mRNA expression was detected by exon array and real-time PCR at 48 h after transfection with miR P-27-5p or NC mimetics and normalized against that of *GADPH*. (**D**) CDK4 protein, (**E**) Phosphorylated RB1, and RB1 proteins were measured by Western blot at 48 h after transfection with miR P-27-5p or NC mimetics. β-actin was used as the internal control. All the experiments were conducted in triplicates.

From exon array and network analysis, we found CDK4 is important in miR P-27-5p–regulated function. We used the sequence alignment method to examine whether *CDK4* is a potential target of miR P-27-5p. Figure 5A shows that the sequence of miR P-27-5p is complimentary to the 3′-UTR region of *CDK4*. To investigate whether miR P-27-5p directly recognizes the 3′-UTR of *CDK4*, we cloned the fragment containing the presumed target site into 3′-UTR of the luciferase gene. Figure 5B shows that transfection with miR P-27-5p resulted in a significant decrease in luciferase activity compared to that in NC-transfected cells. To validate *CDK4* as a target of miR P-27-5p, downregulation of *CDK4* at the mRNA and protein level was examined by exon array, real-time PCR, and Western blot analyses (Figure 5C,D). *CDK4* mRNA and protein in miR P-27-5p–transfected cells were decreased compared to NC-transfected cells. These results suggest that *CDK4* is the functional target of miR P-27-5p in T-47D cells and that miR P-27-5p inhibits cell proliferation and causes cell cycle arrest via targeting *CDK4*.

### 2.5. miR P-27-5p Inhibits RB1 Phosphorylation

To investigate the association of the miR P-27-5p–induced G1 arrest with RB1 phosphorylation, Western blot analysis was conducted using an RB1 phosphospecific antibody. The results showed a decrease in RB1 phosphorylation after transfection with miR P-27-5p (Figure 5E). When dephosphorylated, RB1 interacts with E2F transcription factors and prevents transcription of genes required for progression through the cell cycle. By contrast, when phosphorylated by cell cycle-dependent kinases like CDK2 and CDK4, RB1 no longer interacts with E2F and the cell cycle proceeds through the G1/S checkpoint [17]. Loss of cell cycle control is a hallmark of cancer, and aberrations in the cyclin-CDK-RB pathway are common in breast cancer [25]. Therefore, targeted inhibition of CDK4 activity has a role in the treatment of breast cancer [14,26].

### 2.6. Discussion

Our previous report suggested that miR P-27-5p may be an alternative mature form of miR-802 [6]. Our unpublished data indicate that miR P-27-5p is expressed higher in MCF-10A than MCF-7 and T47D tumor cells. However, previous study reported endogenous miR-802 was not detected in breast cancer patients based on miRNA expression profiles [27]. On the other hand, miR-802 was investigated in mice by using expression and sequencing studies [28] and Kuhn *et al.* used miRNA expression profiling, miRNA RT-PCR and miRNA *in situ* hybridization experiments to identify miR-802 was up-regulated in fetal brain and heart specimens from individuals with Down syndrome when comparing with control groups [29]. According to these, miR-802 is expressed in other tissues, but not in breast tissues. Thus, the expression levels of miR P-27-5p and miR-802 in breast tissues are different.

Our previous study showed that miR P-27-5p could target Laminin β3 (LAMB3) [6]. LAMB3 has been detected in various cancers and affects tumor progression. For example, promoter demethylation of LAMB3 causes aberrant LAMB3 expression in gastric cancer and tumor cells stably expressing LAMB3 have elevated migration and adhesiveness [30]. Patients with esophageal carcinoma have worse 5-year survival rates [31]. Additionally, LAMB3 overexpression promotes migration of prostate cancer LNCaP cells and tumor growth in mice [32]. Thus, these results indicate that LAMB3 is involved in tumor progression, such as tumor growth and metastasis, and miR P-27-5p may affects tumor progression through regulating LAMB3 expression.

From our exon array, network analysis, and functional enrichment analysis, we predicted that miR P-27-5p may have a function in cell cycle and cell proliferation. With cell viability assay and cell cycle analysis, we further confirmed that miR P-27-5p induces cancer cell cycle arrest at the G1 phase and inhibits cancer cell proliferation. De Guire *et al.* find that artificial miRNAs reproduce the effects of E2Fs inhibition in both normal human fibroblasts and prostate cancer cells, where they inhibit cell proliferation and induced cellular senescence [33]. Brown and Naldini suggest utilizing artificial miRNA target sites to exploit or inhibit endogenous miRNA regulation for therapeutic and experimental applications [34]. Idogawa *et al.* efficiently used a single recombinant adenovirus expressing p53 and p21-targeting artificial miRNAs to induce apoptosis in human cancer cells [35]. Collectively, artificial miR P-27-5p may represent a novel therapeutic option in breast cancer treatment.

Previous studies showed that CDK4 regulates progression through the G1/S phase of the cell cycle by binding to cyclin D1 to phosphorylate RB1 and releasing E2F transcription factors for progression through the cell cycle [36,37]. Decreased cyclin D1 and cyclin D1-CDK4/6 kinase activity reduces the invasion and migration potential of MDA-MB-231 breast cancer cells [37]. In this present study, functional enrichment of miR p-27-5p-regulated network showed that CDK4 is involved in many biological functions such as posttranslational protein modification, protein amino acid phosphorylation, phosphorylation, cellular protein metabolic process, positive regulation of cellular process, protein modification process, regulation of cell cycle, regulation of cell proliferation, and interphase (Table 1). We found that CDK4 and RB1 were hubs within this network and RB1 was one of the interacting proteins of CDK4. RB1 is involved in positive regulation of cellular process, regulation of cell cycle, regulation of cell proliferation, and interphase. These results are consistent with the reports described by Prud’homme *et al.* [36] and Zhong *et al.* [37]. Furthermore, we identified their expression levels of CDK4 and phosphorylated RB1 were reduced in response to miR p-27-5p over-expression. Thus, these results indicate that miR p-27-5p may play important roles in cell cycle and proliferation by regulating a small subset of genes within its regulated PIN.

In this study, artificial miR P-27-5p targets the 3′-UTR of *CDK4* with mismatches to the central region of its targeting site (Figure 5A). Ye *et al.* designed artificial miRNAs (AmiRs) with mismatches targeting the 3′-UTR of viral genome. These small AmiRs combined with pRNA-folate conjugates could form a promising system for antiviral drug development [38]. Furthermore, we found that miR P-27-5p suppresses the expressions of CDK4, both in mRNA and protein levels (Figure 5C,D), and inhibits RB1 phosphorylation (Figure 5D), which is one of the CDK4-interacting proteins (Figure 1A). As described previously, CDK4 regulates progression through the G1/S phase of the cell cycle by binding to cyclin D1 to phosphorylate RB1 [36,37]. All of the evidence implies that the miR P-27-5p–induced cell cycle and inhibited cell proliferation may be through targeting CDK4 and its interacting protein, RB1.

## 3. Experimental Section

### 3.1. Cell Culture and Transfection

We used one human mammary epithelial (normal) cell line (MCF-10A) and three breast cancer cell lines (MCF-7, T-47D) in this study. All the cell lines were purchased from the Bioresource Collection and Research Center, Hinchu, Taiwan. Human breast MCF-10A cells were cultured in DMEM/F12 supplemented with 5% horse serum and MCF-7 and T-47D cells were cultured in DMEM supplemented with 10% FBS and 100 μg/mL penicillin/streptomycin at 37 °C in a humidified atmosphere of 5% CO_2_. miR P-27-5p mimics and their corresponding NC were obtained from Ambion (Applied Biosystems/Ambion, Austin, TX, USA). Transfection was carried out using Lipofectamine 2000 (Invitrogen, Inc.) according to the manufacturer’s instructions [6].

### 3.2. Exon Arrays and Data Analysis

Total cellular RNA was extracted with the use of TRIzol reagent (Invitrogen), and purity was confirmed by spectrophotometry (A_260_/A_280_ ratio) and capillary electrophoresis (Agilent 2100 Bioanalyzer, Agilent). RNA processing and hybridization onto Affymetrix Human Exon 1.0 ST arrays were performed according to the manufacturer’s protocol. Microarray (*n* = 2 per group) analysis was performed by dChip software (dChip 2010.01; Cheng Li Lab of Computational Cancer Genomics: Boston, MA, USA, 2010) [39]. Raw data (CEL files) and annotation data were used for computing the signal value of exons with Quantile normalization. The exon signal data were later transformed to the Affymetrix U133 2.0 PLUS probe set by the export function of dChip software to obtain the gene expression value for further analysis. We have submitted the exon array data to the GEO database, and the series record is GSE28657.

### 3.3. Protein Interaction Network and Functional Enrichment

Among the significantly differentially expressed genes (*p* < 0.05), the downregulated genes were chosen due to possible miR p-27-5p targets. The protein interaction networks with these downregulated genes were constructed based on human PIN from the Human Protein Reference Database (version 9; Institute of Bioinformatics & Pandey lab: Bangalore, India; Baltimore, MD, USA, 2010). The possible functions of this network were analyzed by GO functional enrichment, which were performed by BiNGO with a threshold of *p* < 0.001 and a GO level of more than 5 [20]. Enriched functions with the CDK4 gene were selected for further discussion. The method was described previously [20].

### 3.4. Cell Viability Assay

Cells (2 × 10^4^ cells/mL) were seeded on 24-well plates. After 24 h, the cells were transfected with miR P-27-5p (60 nM). At 24 h, 48 h, or 72 h, MTT (100 μL) was added to each 1 mL of culture medium for 4 h of incubation at 37 °C and measured at 570 nm by ELISA reader. All experiments were performed in triplicates.

### 3.5. Cell Cycle Analysis

Cells (4 × 10^5^) were transfected with miR P-27-5p or a control vector at a final concentration of 100 nM using Lipofectamine 2000 (Invitrogen, Inc.) for 48 h. After incubation, floating and adherent cells were harvested. Cells were washed carefully with cold PBS at 4 °C. Cell pellets were resuspended in 100 μL of a binding buffer. Annexin-V FITC (0.2 μg/100 μL) and PI (10 μg/mL) were added to the cells and left to incubate in the dark for 15 min at room temperature. All data were obtained using flow cytometry with a FACSCanto cytometer (Becton Dickinson, San Jose, CA, USA). The flow cytometric analysis was performed using FCS Express (version 4; DeNovo software: Los Angeles, CA, USA, 2012). All experiments were performed in triplicates.

### 3.6. Construction of the CDK4 3′-UTR Report Plasmids and Luciferase Assay

A CDK4 3′-UTR luciferase reporter was created by inserting full-length human CDK4 3′-UTR into the *Sac*I and *Hind*III sites in the pMIRREPORT luciferase expression vector (Ambion), as described before [6]. Specific fragments, including the miR P-27-5p targeting site of CDK4 3′-UTR, were generated by the primers: 5′-GGG GAG CTC GTT ACC TCA CCG ACG GTA CCT T-3′ (forward) and 5′-GGG AAG CTT CTG GTA ATA AAG AAA CAA AAC-3′ (reverse). Clones were selected after colony PCR and restriction enzyme digestion. The clones were verified by sequencing (Mission Biotech Co., Ltd.). All the restriction enzymes were purchased from New England Biolabs. Reporter activity was assayed using the Dual-Light system (Applied Biosystems) and was normalized to β-galactosidase activity to control for transfection efficiency variation among different wells according to the manufacturer’s instructions. Luminescent signal was quantified by the Spectramax M5 ELISA reader (Molecular Devices). All experiments were performed in triplicates.

### 3.7. Quantitative Real-Time PCR

Total RNA was isolated from T-47D cells, and first-strand cDNA synthesis was generated. Extracted first-strand cDNAs were analyzed using a BioRad iCycler iQ Real-Time Detection System with the SYBR Green dye (Molecular Probes, Eugene, OR, USA). SYBR Green yields a strong fluorescent signal on binding double-stranded DNA, enabling the quantification of gene expression by measurement of the intensity of the fluorescent light. The mRNA expressions of CDK4 were normalized to RNA content for each sample by using GADPH gene products as internal controls. The relative expression levels were calculated as the ratio of expression from miR P-27-5p transfected cancer cells to the control. All reactions were run in triplicates. The sequences of the primers specific to CDK4 were: forward primer, 5′-CTCTGCGTCCAGCTGCTCCG-3′; and reverse primer, 5′-ATCAAGGGAGACCCTCACGC-3′.

### 3.8. Western Blot

The protein (20 μg) was loaded on a 10% SDS-PAGE and subsequently transferred onto PVDF membranes (Amersham Biosciences) at 150 V for 1.5 h. The membranes were blocked in 5% nonfat milk in PBST containing 0.1% Tween 20, incubated with primary antibody overnight at 4 °C, and followed by incubation with a secondary antibody (a goat antirabbit-conjugated IgG, 1:10000 dilution, Upstate). CDK4, phospho-RB1, RB1, and β-actin were purchased from Cell Signaling, Epitomics, Epitomics, and Millipore, respectively. After incubation with secondary antibodies, immunoblots were visualized with the ECL detection kit (Amersham Biosciences) and exposed to x-ray film. The protein bands were quantified using ImageMaster software version 6.0 (Amersham Pharmacia Biotech, Geneva, Switzerland, 2005), and the data were normalized to β-actin.

### 3.9. Statistical Analysis

Statistical analysis was performed using Microsoft Office Excel 2007 (Microsoft Corporation: Redmond, WA, USA, 2007). Student’s *t* test and *P*-values were tested for multiple comparisons by controlling the false discovery rate. Changes were considered significant if the false discovery rate was less than 0.05.

## 4. Conclusions

Our results reveal that miR P-27-5p targets and negatively regulates CDK4, which in turn suppresses RB1 phosphorylation, thereby preventing the progression of cancer cell cycle and promoting G1 arrest. As a consequence, cancer cell proliferation is inhibited. Thus, on the therapeutic side, artificial miR P-27-5p has the potential for the application in the treatment of breast cancer. miRNA can affect many downstream targets, which in turn form a complicated network. Therefore, there is considerable potential for the miRNA-regulated network to be a novel therapeutic target. The therapeutic strategy could be used for cancer as well as the other diseases such as neurological and cardiovascular disorders. RNA interference and miRNA oligonucleotides have clinical potential. In the future, using the miR P-27-5p-regulated network as a novel therapeutic target, especially responsible for G1 arrest, may help reduce off-target effects and alleviate the risk of side effects.

## Supplementary Material



## Figures and Tables

**Table 1:** The functions and genes regulated by miR P-27-5p.

Description	Gene List	*p*-Value	Adj. *p*-Value
protein modification process (GO:0006464)	*STK36 STYX USE1 EIF5A CASK RPS6KB2 AURKB MAP3K6 MAP3K5 ERO1LB ST3GAL3 TRAK1 IFNG CLK4 LOX INSR SYK EGFR DBNL BCR TNIK PTPRF DTL WNK1 PTPRS PIM2 HDAC11 SUV420H2 PPP1CA HUWE1 MAPK3 AMFR UBA52 TNFAIP1 GGCX FGFR4 ERBB3 STK11 ERBB2 MAPKAPK3 NEDD8 ANAPC11 EPHB3 ACP1 SRC EPHB6 UBE2D2 PTK6 PRKRA GNMT TRAF7 MGAT4B B4GALT1 P4HB MAP2K2 TKT HDAC5 CDC42BPG HDAC3 PLK1 DOHH GRK6 SPTBN1 EAF2 MERTK HDAC6 BCKDK FASTK UCHL1 CAD AKT1 PAK7 PIGM PRMT2 ASH2L HSF1 PAK4 TGFA RAB6A KLHL20 CTBP1 PIGX DDB1 PIGU PIGT CDK6 UBE2I CDK4 PRKCE CDK2 SRPK1 G6PD BTG1 DDB2 DHPS SERP1 PRKCZ PPP4R2 RAB3D UBE3A USP5 GPAA1 ABI2 STUB1 IGF1R STT3A CDC2L2 PER1 C19ORF62 PRKAA2 PPP3CA THBS1 YES1 QSOX1 KAT2A PDK2 LRSAM1 FLT1 FLT4 SIRT2 RPS6KA1 MAPK13 CSNK1E RSRC1 MAPK15 ARAF JAK1 DPM2 DPM3 IRF4 RBM14 RNF40*	7.98 × 10^−8^	6.34 × 10^−6^
protein amino acid phosphorylation (GO:0006468)	*BCKDK FASTK STK36 CASK RPS6KB2 CAD AURKB AKT1 PAK7 MAP3K6 MAP3K5 HSF1 PAK4 IFNG CLK4 TGFA INSR SYK EGFR DBNL CTBP1 BCR TNIK WNK1 CDK6 PIM2 CDK4 PRKCE CDK2 SRPK1 MAPK3 PRKCZ FGFR4 ERBB3 STK11 ERBB2 MAPKAPK3 ABI2 EPHB3 SRC IGF1R EPHB6 CDC2L2 PTK6 PRKRA PRKAA2 THBS1 YES1 PDK2 FLT1 MAP2K2 FLT4 TKT CDC42BPG RPS6KA1 MAPK13 CSNK1E PLK1 RSRC1 MAPK15 ARAF GRK6 JAK1 SPTBN1 MERTK*	3.24 × 10^−6^	1.43 × 10^−4^
phosphorylation (GO:0016310)	*BCKDK UQCRC1 FASTK STK36 SNCA CASK RPS6KB2 CAD AURKB AKT1 PAK7 MAP3K6 MAP3K5 HSF1 PAK4 IFNG CLK4 TGFA INSR SYK EGFR DBNL CTBP1 BCR TNIK WNK1 CDK6 PIM2 CDK4 PRKCE CDK2 SRPK1 ATP6V1A MAPK3 PRKCZ FGFR4 NDUFB8 ERBB3 STK11 ERBB2 MAPKAPK3 ABI2 ATP6V1B1 EPHB3 SRC IGF1R EPHB6 CDC2L2 PTK6 PRKRA PRKAA2 THBS1 YES1 PDK2 ATP5J2 FLT1 MAP2K2 FLT4 TKT CDC42BPG RPS6KA1 MAPK13 PLK1 CSNK1E RSRC1 MAPK15 ARAF GRK6 JAK1 SPTBN1 MERTK*	2.66 × 10^−5^	9.24 × 10^−4^
regulation of cell proliferation (GO:0042127)	*FGF18 EIF5A GJA1 STRN SSR1 MEN1 MAGED1 PGR BNIPL CDKN2A ASH2L HSF1 APOE CDKN2D HEY2 IFNG TGFA RARB DLG5 ODZ1 NR2F2 INSR LTA SYK EGFR CTBP1 CAPNS1 PTPRF DTL RXFP2 HTR4 ESR1 CDK6 ESR2 RB1 CDK4 RPS4X CDK2 RBBP9 MXD4 NME2 BTG1 NME1 DHPS ATPIF1 SMARCA2 CAV2 PRKCZ DERL2 FGFR4 MVD TBC1D8 ERBB3 STK11 FOXM1 ERBB2 BCL2L1 MIF IGF1R PRKRA ADRA2A RNF10 BCL6 THBS1 TINF2 EMD B4GALT1 FLT1 NF2 CREB3 TAF6 FLT4 CSNK2B TKT FZD7 CAPN1 TSC2 GLMN PBX1 ADRA1D IGFBP5*	8.40 × 10^−7^	4.35 × 10^−5^
post-translational protein modification (GO:0043687)	*STK36 STYX USE1 CASK RPS6KB2 AURKB MAP3K6 MAP3K5 ERO1LB IFNG CLK4 INSR SYK EGFR DBNL BCR TNIK PTPRF DTL WNK1 PTPRS HDAC11 PIM2 SUV420H2 PPP1CA HUWE1 MAPK3 AMFR TNFAIP1 GGCX FGFR4 ERBB3 STK11 ERBB2 MAPKAPK3 NEDD8 ANAPC11 EPHB3 ACP1 SRC EPHB6 UBE2D2 PTK6 PRKRA TRAF7 MAP2K2 TKT HDAC5 CDC42BPG HDAC3 PLK1 GRK6 SPTBN1 EAF2 MERTK HDAC6 BCKDK FASTK UCHL1 CAD AKT1 PAK7 PRMT2 HSF1 ASH2L PAK4 TGFA RAB6A KLHL20 CTBP1 DDB1 PIGU PIGT UBE2I CDK6 PRKCE CDK4 SRPK1 CDK2 BTG1 DDB2 PRKCZ RAB3D UBE3A USP5 GPAA1 ABI2 STUB1 IGF1R CDC2L2 C19ORF62 PRKAA2 PPP3CA THBS1 YES1 QSOX1 KAT2A PDK2 LRSAM1 FLT1 FLT4 SIRT2 RPS6KA1 MAPK13 CSNK1E RSRC1 MAPK15 ARAF JAK1 IRF4 RBM14 RNF40*	2.96 × 10^−7^	1.96 × 10^−5^
cellular protein metabolic process (GO:0044267)	*USE1 RPLP2 AURKB CCT3 MAP3K6 MAP3K5 ERO1LB RPLP0 RPLP1 IFNG CLK4 TRAK1 FAU RPL10 PSENEN SYK DBNL BCR TNIK PTPRF WNK1 PTPRS PIM2 SUV420H2 PPP1CA RPS19 HUWE1 BACE2 BACE1 MAPK3 AMFR GGCX ERBB3 RAD23A ERBB2 EPHB3 ACP1 EPHB6 RPS28 MGAT4B RPSA MAP2K2 TKT RPS6 RPS7 CCT7 CDC42BPG RPL18A GRK6 BCKDK NARS FASTK QARS CANX HSF1 ASH2L TGFA RAB6A CTBP1 SRPK1 G6PD EEF1G EEF1D SERP1 TUFM RAB3D USP5 GPAA1 ABI2 RPL36 STT3A MRPL12 PER1 C19ORF62 PRKAA2 PPP3CA THBS1 QSOX1 PDK2 LRSAM1 EIF2B1 RPL28 RPL29 RPS6KA1 CSNK1E RPL22 PSMD11 MAPK13 ARAF MAPK15 DPM2 DPM3 RBM14 STK36 STYX EIF5A CASK RPS6KB2 ST3GAL3 LOX INSR RPS27A EGFR DTL EEF2 HDAC11 UBA52 TNFAIP1 FGFR4 DERL2 EEF1B2 STK11 MAPKAPK3 NEDD8 ANAPC11 DERL3 SRC EIF3D UBE2D2 PSMB6 EIF3H RPL6 PTK6 PSMB3 EIF3F PRKRA RPL8 EIF3K EIF3L GNMT TRAF7 B4GALT1 P4HB EEF1A1 SEC11A EIF4B HDAC5 ATF6 HDAC3 PPIA DOHH PLK1 PSMC3 SPTBN1 DNAJB1 EAF2 MERTK HDAC6 COPA UCHL1 CAD AKT1 PAK7 PRMT2 PIGM PAK4 PSMD2 KLHL20 PIGX DDB1 PIGU PIGT UBE2I CDK6 PRKCE RPS4X CDK4 CDK2 BTG1 TBCD DDB2 DHPS PRKCZ PPP4R2 FKBP8 APH1A UBE3A FKBP4 APH1B STUB1 IGF1R CDC2L2 LARS YES1 KAT2A FLT1 FLT4 SIRT2 NCSTN RPL13A RSRC1 JAK1 IRF4 RNF40*	4.47 × 10^−13^	1.78 × 10^−10^
positive regulation of cellular process (GO:0048522)	*MMS19 XRCC5 FGF18 THRA SNCA UTRN RPS6KB2 CASK EIF5A ITSN1 SSR1 MAGED1 PGR MAP3K5 CDKN2A HTRA2 CD44 PACSIN3 APOE IFNG PSENEN RARB INSR SYK EGFR PLD2 CAPNS1 PTPRF PCBD1 DFFA DTL HTR4 MED12 PIM2 TBR1 NCAM1 SPAG9 NME2 RPS19 NME3 PIAS3 NME1 VAMP3 SMARCA2 SMARCA4 CAV2 DERL2 FGFR4 MVD ERBB3 PFKFB2 GLUD1 ERBB2 RRM2B ANAPC11 BCL2L1 TUBB PSMB6 ARPC2 SMARCB1 PSMB3 P2RY2 NUMB PRKRA ADRA2A RNF10 TRAF7 TRAF4 B4GALT1 DVL2 TP53BP1 TEAD1 TKT TPD52L1 RPS6 SREBF2 ATF6 TRAF3IP2 HDAC5 ATF4 PSMC3 PLK1 IKBKG GLMN RFX3 PDZK1 PDCD6 ADRA1D PC HDAC6 E2F1 RTN4 LZTS2 FASTK GJA1 PPOX RFXANK MEN1 AKT1 BNIPL FOS CCNE1 ASH2L MAPT HEY2 PSMD2 TGFA RHOC NR2F2 CASP2 LTA ZFP36 RXFP2 ESR1 CDK6 ESR2 RB1 PFKM CDK4 RPS4X PRKCE HMGA1 CDK2 FLNA PRPF6 SCAP NCOA3 BTG1 IGF2R SORT1 DHPS EEF1D C6ORF108 BID MBL2 PRKCZ APH1A TBC1D8 UBE3A USP5 FOXM1 APH1B TRAIP STUB1 CALCOCO1 MIF IGF1R MRPL12 SOS2 C19ORF62 BCL6 THBS1 TINF2 TERF1 PHLDA1 FLT1 NF2 CREB3 PSAP FLT4 MAP1B BIRC5 DPYSL2 FZD7 CAPN1 NCSTN PKNOX1 PSMD11 CSNK1E MAPK15 BRE PBX1 IRF4 RBM14 DNM2*	1.41 × 10^−10^	2.52 × 10^−8^
interphase (GO:0051325)	*E2F1 EGFR TPD52L1 BIRC5 CDK6 ZNF655 RB1 CDK4 RPS6 CDK2 AKT1 CCNE1 SIN3A CDKN2A CDKN2D PPP3CA TERF1 DNM2*	1.90 × 10^−5^	6.87 × 10^−4^
regulation of cell cycle (GO:0051726)	*E2F1 E2F2 CDT1 MEN1 AKT1 GSS CDKN2A CLP1 CDKN2D IFNG TGFA FANCG NR2F2 INSR EGFR BCR DTL DDB1 CDK6 RB1 TACC3 CDK4 CDK2 RAD1 CHMP1A SGSM3 PRNP MAD2L2 DST SMARCA4 CAV2 STK11 FOXM1 ZNF655 CDC37 MIF BCL6 C19ORF62 THBS1 TERF1 TAF6 CREB3 BIRC5 RPS6 SIRT2 HDAC3 PLK1 TSC2 CKS2 BRE DGKZ*	6.92 × 10^−7^	3.82 × 10^−5^
